# Roles of glycosylation at the cancer cell surface: opportunities for large scale glycoproteomics

**DOI:** 10.7150/thno.81760

**Published:** 2023-04-23

**Authors:** Tomislav Čaval, Frederico Alisson-Silva, Flavio Schwarz

**Affiliations:** InterVenn Biosciences, South San Francisco, California.

**Keywords:** Cancer, Mass Spectrometry, Glycosylation, Glycoproteomics, Immune checkpoints

## Abstract

Cell surface glycosylation has a variety of functions, and its dysregulation in cancer contributes to impaired signaling, metastasis and the evasion of the immune responses. Recently, a number of glycosyltransferases that lead to altered glycosylation have been linked to reduced anti-tumor immune responses: B3GNT3, which is implicated in PD-L1 glycosylation in triple negative breast cancer, FUT8, through fucosylation of B7H3, and B3GNT2, which confers cancer resistance to T cell cytotoxicity. Given the increased appreciation of the relevance of protein glycosylation, there is a critical need for the development of methods that allow for an unbiased interrogation of cell surface glycosylation status. Here we provide an overview of the broad changes in glycosylation at the surface of cancer cell and describe selected examples of receptors with aberrant glycosylation leading to functional changes, with emphasis on immune checkpoint inhibitors, growth-promoting and growth-arresting receptors. Finally, we posit that the field of glycoproteomics has matured to an extent where large-scale profiling of intact glycopeptides from the cell surface is feasible and is poised for discovery of new actionable targets against cancer.

## Introduction

Glycans are intimately involved in almost every biological process 1. Perhaps the most striking is their role in cancer development where cellular and metabolic changes during oncogenic transformation lead to the expression of aberrant glycosylation on the cell surface. Consequently, aberrant glycosylation has been associated with the acquisition of all the cancer hallmarks 2; such as enhancing cell proliferation through regulation of growth factor receptors, resistance to cell death through modification of cell death receptors, and escape from immune responses through alteration of the stability of ligands of immune checkpoint receptors. The study of protein glycosylation has been marred by technical challenges in the past. Recently, mass spectrometry has risen as one of the most powerful approaches to study protein glycosylation. For example, analysis of released glycans has reached a stage where high throughput, sensitivity, accuracy, and reproducibility is commonly achieved, enabling detailed-insight into cancer glycosylation 3-5. However, with the rising awareness of the roles of glycosylation on specific proteins and glycosylation sites there is a pressing need to reach the same level for intact glycopeptide analysis. While such an approach is potentially more informative, high entry barriers prevented widespread acceptance. Recently, through large scale community efforts great strides have been made in software development, making glycoproteomics more accessible 6-9. Moreover, collection of sophisticated methods of glycopeptide enrichment 10,11 and fragmentation 11,12 are enabling large-scale glycoproteomic analysis of cells, tissues and bodily fluids.

In this review, we focus on the importance of the structure-function nexus for glycoproteins on the surface of cancer cells: we describe examples of broad glycosylation changes observed in cancer and highlight their function on selected proteins involved in cancer development, progression and immune evasion. We then posit that large-scale intact glycopeptide profiling from the cell surface is well within reach, unraveling a new layer of biological information and promising new targets for development of more effective therapies. While our review predominantly focused on *N*-glycosylation, mainly due to technological maturity, it is worth pointing out that other types of glycosylation are also being increasingly explored (for a review, see 13-16).

## Broad glycosylation changes at the surface of cancer cells

Numerous studies have implicated aberrant glycosylation on cancerous cells leading to the development of their metastatic and invasive potential 17-23. Cancer cells rewire their metabolism to promote growth, survival, proliferation, and long-term maintenance. A common feature of cancer cells is an increase in glucose uptake and its fermentation to lactate even in the presence of oxygen, a metabolic rewire known as Warburg effect that sustains the high energy demand of cancer cells 24. The increased glycolysis influx in tumor cells is followed by increased flux through the hexosamine biosynthetic pathway (HBP), a branch of glycolysis that is initiated by the rate limiting enzyme Glutamine: Fructose-6-phosphate aminotransferase (GFAT) 25. The HBP is responsible for the production of UDP-N-acetylglucosamine (UDP-GlcNAc), an activated monosaccharide required for N-glycosylation that also serves as metabolic precursors for UDP-GalNAc and CMP-Neu5Ac 26, therefore strongly impacting cell surface glycosylation during cancer 27.

Hypersialylation of cancer cells results from either altered sialyltransferase substrate availability, as mentioned above, or altered levels of sialyltransferases and neuraminidases 28. Hypersialylation generally increases ligands for sialic acid-binding immunoglobulin type lectins (Siglecs), which are commonly expressed on the surface of immune cells 29. Hypersialylated glycans from tumor cells can engage Siglecs expressed in dendritic cells^30^, natural killer cells 31, tumor associated macrophages 32 and CD8+ T cells 33 promoting immunosuppressive signaling, and endowing cancer with the ability to avoid detection and removal by the immune system. Recent studies have put Siglecs on the roadmap as potential targets in cancer immunotherapy 34-37 and introduced antibody-sialidase conjugates to remove Siglec ligands and improve immune recognition 38,39. In a proof of principle study, trastuzumab-sialidase conjugate overcome antibody dependent cell cytotoxicity (ADDC) resistance in breast cancers with low HER2 levels 38.

Besides sialylation, an increase in fucosylation is also a common feature in cancer development and progression. Fucosyltransferase 8 (Fut8), the enzyme responsible for core fucosylation, shows low expression in healthy liver and is markedly overexpressed in cancer. Recently, a matched characterization of glycosylation between primary and metastatic melanoma patient derived tissues has been described, revealing FUT8 as a driver of melanoma metastasis 40. Mechanistically, the study demonstrated that TGFβ-Induced Factor Homeobox 2 (TGIF2) regulates FUT8 transcription which add core fucose to neural cell adhesion molecule, L1CAM, resulting in altered L1CAM activity and/or interactions with other growth factor receptors. This is likely only the tip of the iceberg of unexplored biology.

In addition to its role during TGFβ signaling, fucosyltransferases are also involved in the synthesis of the carbohydrate antigens sialyl Lewis a (sLe^a^) and sialyl Lewis x (sLe^x^), terminal N-glycan tetrasaccharide structures composed of Siaα2-3Galβ1-3(Fucα1-4)GlcNAc and Siaα2-3Galβ1,4(Fucα1-3)GlcNAc, respectively. sLe^x^ is constitutively expressed on the cell surface of leukocytes and is the minimum epitope required for leukocyte engagement to E-selectins expressed on endothelial cells during leukocyte rolling^41^. However, both sLe structures have been frequently observed in a number of cancers and are known to influence metastatic potential 42,43 by facilitating tumor cell interaction with selectins expressed on leukocytes, platelets and endothelium 44. The expression of sLe^x^ in estrogen receptor alpha-positive breast cancers correlated with bone metastasis, and both sLe^x^ and sLe^a^ expression correlated with metastasis and poor survival in colorectal cancer 43,45,46. These observations have proven useful in development of biomarkers for diagnosis of tumor burden (i.e, sLe^a^, known as CA19-9, in pancreatic cancer). In fact, the introduction of glycosyltransferase genes leading to the expression of sLe^a^ antigen in mice that otherwise lack it led to pancreatitis and pancreatic cancer 47. In this study CA19-9 modification of the matricellular protein Fibulin 3 in mice increased its interaction with EGFR resulting in hyperactivation of this signaling pathway. This work undoubtedly paves the way for using sLe^a^ as an actionable therapeutic target, and indicates that altered glycosylation could be causative as opposed to being merely correlative with disease.

Lastly, one intriguing epitope gaining recognition is the presence of bisecting GlcNAc on *N*-glycans, catalyzed by GlcNAc-T III enzyme encoded by *Mgat3* gene that has been reported to be immunosuppressive almost 30 years ago 48,49. For example, bisecting GlcNAc *N*-glycans are thought to play a key role in human sperm evading maternal immune response. In cancer, however, the dominant role of bisecting GlcNAc appears to be suppression of terminal N-glycan modifications 50.

## Functional consequences of aberrant glycosylation at a protein level

While changes of the cell surface glycosylation in cancer are well described, less is known about whether such changes are simply correlative of cancer or are causative in neoplasia. Similarly, little is known about functional pathways that may be controlled by aberrant glycosylation. In this section, we focus on selected examples where aberrant *N-*glycosylation directly influences the underlying protein, and consequently downstream signaling pathways in cancer. While literature is riddled with examples of glycosylation influencing protein function in cancer, and we point the readers to a comprehensive recent review 51, it can broadly be ascribed to glycosylation changes on growth-promoting and/or growth-arresting cell surface receptors. A landmark study demonstrated that *N*-glycan multiplicity, which means the number of *N*-glycosites on a given protein backbone, is in general higher for growth-promoting receptors and lower on growth-arresting receptors 52. The *N*-glycan multiplicity serves as a sensor of intracellular glucose metabolism and can be regulated to either reduce cell growth by lowering the cell surface levels of *N*-glycans or to increase cell growth via increasing *N*-glycan amount 52,53. In the next part we briefly cover recent examples of specific glycans influencing function of cell surface proteins.

Epidermal growth factor receptor (EGFR), a receptor tyrosine kinase with 12 potential *N*-glycosylation sites, is perhaps the most famous example of functional influence of glycosylation on a cell surface protein 54. While EGFR core fucosylation promotes receptor dimerization 55, the presence of the antennary α1-3 (sLe^X^) fucose or increased EGFR sialylation have been shown to attenuate EGFR dimerization and signaling 56. Additionally, the ST6GAL1 catalyzed α2-6 sialylation of EGFR was shown to confer resistance to gefitinib, an EGFR inhibitor 57. Intriguingly, a simple switch of antennary fucose from α1-3 (sLe^X^) to α1-4 (sLe^A^) seems to be associated with hyperactivation of EGFR in gastric cancer, and can be therapeutically targeted with anti-sLe^A^ antibody 58. Recently, presence of bisecting-GlcNAc was shown to also attenuate EGFR signaling and decrease migratory and proliferative potential of breast cancer cells 59. Finally, in addition to forming homodimers to enable downstream signaling, EGFR is also known to form heterodimers and there are preliminary reports demonstrating that specific glycan-glycan interactions can drive their formation 60. In fact, recent work has implicated α2,6-sialylated N-glycans of a known dimerization partner of EGFR, human epidermal growth factor receptor 2 (ErbB2/HER2), as a driver of trastuzumab resistance in gastric cancer^61^,^62^. Mechanistically a removal of α2,6-sialylated *N*-glycans from ErbB2 via silencing of sialyltransferase ST6GAL1 leads to increase of α2,3-sialylation, and terminal fucosylation, resulting in stabilization of ErbB2 dimers at the cell surface rendering them more sensitive o trastuzumab induced cytotoxicity. The authors of this study also discuss the possibility of glycosylation decreasing ErbB2's ability to form heterodimers with EGFR and downregulating EGFR activation following trastuzumab treatment^62^.

Vascular endothelial growth factor receptor-2 (VEGFR2), another receptor tyrosine kinase with 18 potential *N*-glycosylation sites, regulates the relationship between tumor cells and endothelium. VEGFR signaling is upregulated by tumor hypoxia promoting angiogenesis aiding the tumor cells into and out of the blood stream 63,64. Moreover, it is also known that hypoxic microenvironments can increase N-glycans branching of VEGFR2 and that this glycan remodeling makes tumor vasculature resistant to anti-VEGF treatment. This hypoxia-promoted increase in VEGFR2 *N*-glycan branching leads to a stronger interaction between Galectin 1 and endothelial cells, resulting in VEGFR2 activation in a ligand-independent manner. In addition, a follow up study demonstrated that the capping of *N*-glycans at Asn-247 of VEGFR2 by sialic acid, tunes ligand-dependent activation and signaling of VEGFR2 in endothelial cells 65. By using in-depth glycomics, glycoproteomics, and functional profiling, the authors revealed that the capping of the Asn-247 N-glycan with sialic acid impairs receptor function while the absence of sialic acid units leads to its activation 65. This is very similar to the observations described in previous paragraph on the role of glycans in EGFR signaling except for fucosylation, which appears not to play a role in VEGFR2 activation.In the case of growth-arresting receptor, it is noteworthy how core fucosylation of the Transforming growth factor beta receptor 1 (TGFβR1) is required for its function. Here, FUT8 catalyzed addition of fucose in α-1,6-linkage to core *N*-acetylglucosamine of *N*-glycans remodels TGFβ receptor fucosylation and promotes EMT in breast cancer cells^66^. Similarly, the TGFβR antennary fucosylation mediated by FUT3 and FUT6 also promotes EMT in human colorectal cancer cells^67^. Lastly, intraperitoneal injection of exogenous TGF-β1 in *FUT8 null* mice rescued the emphysema-like phenotype displayed in the lung of these mice^68^, representing one of the first examples of glycoprotein specific rescue in a glycosylation deficient animal model. Next, the presence of bisecting GlcNAc was shown to inhibit TFG-β1 induced epithelial to mesenchymal transition (EMT) 69, again likely due to N-glycan bisection inhibiting poly-*N*-Acetyllactosamine (polyLacNAc) elongation and interaction with galectin-3, which stabilizes surface expression TGFβR 70,71.

Finally, one class of interesting molecules where glycosylation is shown to play crucial role are the members of Immunoglobulin-like immunosuppressive molecules, also known as checkpoint inhibitors, such as programed death ligand 1 and 2 (PD-L1, PD-L2) and cytotoxic T-lymphocyte associated protein 4 (CTLA4) 72. A monoclonal blocking antibody targeting the glycosylated form PD-L1 in breast cancer cells blocked PD-L1/PD-1 interaction by promoting PD-L1 internalization and degradation 72 therefore inducing a potent tumor cell killing effect. The glycosylation of PDL-1 in the triple negative breast cancer cells was shown to be induced by EGFR signaling-mediated upregulation of β-1,3-N-acetylglucosaminyl transferase (B3GNT3), an enzyme catalyzing the addition of polyLAcNAc repeats to PDL-1. The presence of polyLAcNAc on PD-L1 physically stabilizes its interaction with PD-1 causing suppression of T-cell activity. Finally, the authors also utilized this glycosylation signature to develop an antibody drug conjugate specifically binding to polyLacNAc containing PD-L1 that was validated in a syngeneic mouse model of human breast cancer 66. Multiple follow up studies have since exploited the role of PD-L1 glycosylation as a therapeutic target 73-75.

Interestingly, another member of the B7 homolog family 76, PD-L1 and PD-L2 are also known as B7-H1 and B7-DC, respectively, B7-H4 molecule was shown to be stabilized by glycosylation in triple negative breast cancer. Specifically, upregulation of STT3A, an oligosaccharyltransferase required for *N*-glycan transfer to the protein backbone, leads to a higher *N*-glycosylation site occupancy of B7-H4 preventing ubiquitination and subsequent degradation 77. Coincidentally, B7-H3 was shown to be stabilized by core fucosylation and the combination of fucosyltransferase inhibitor, 2F-Fuc, with anti-PDL1 resulted in enhanced therapeutic efficacy in B7-H3 positive triple negative breast cancer 78. This begs the question if glycosylation could be one of the uniting factors behind the B7 family in cancer?

Beyond the B7 family, a landmark work has provided a clue to a 40-year-old mystery on why cell surface desialylation of T-cells leads to an increase in T cell activation 79. Sialylated glycans either on T cells or antigen presenting cells can act as alternative ligands for CD28 competing with CD80(B7-1) binding therefore attenuating co-stimulation. Additionally, since CD28-CD80 axis is one of key pathways to reviving exhausted T cells in immune checkpoint therapy, targeted desialylation could emerge as a powerful way forward in enhancing CD8+ T cell anticancer immune responses 80,81.

Glycosylation influence over the immune system is far beyond these selected few examples. For example, a recent CRISPR activation screening has identified B3GNT2, another enzyme involved in polyLAcNAc elongation, as a key driver of tumor cell resistance to T cell cytotoxicity 82. In this elegant study, the authors show that the presence of polyLAcNAc on around a dozen of cancer cell surface ligands drives resistance against T cell mediated cytotoxicity across several different cancers. However, this is likely only scratching the surface of all the possible ligands carrying polyLAcNAc elongation, a modification that is analytically challenging to identify and position when dealing with thousands of different proteins at once. Altogether, these examples clearly show how the changes in cellular glycosylation can confer plasticity and modulate cell differentiation processes and immune response during cancer. The development of methods to perform in-depth characterization of cell surface glycoproteins, and position the glycosylation to specific sites of the proteins would greatly contribute to understanding the function of the diverse world of glycosylation changes that occur during disease.

## Coming of age of intact cell surface glycopeptide profiling

The isolated examples described above of the role that glycosylation plays in modulating receptors' properties highlight the pressing need of determining the fine picture of the cell surface. What else is hiding on the cell surface? What kind of biology, mechanistic insights and therapeutic targets could we uncover by large scale cell surface glycoproteomics? Even more so, are there any unexpected structures like the recently discovered glycoRNAs that have been hiding in plain sight 83? In this part, we first provide a brief overview of mass spectrometry based glycoroteomics. Next, we summarize the workflows developed for analysis of formerly glycosylated peptides — glycopeptides that have been enzymatically deglycoyslated prior to analysis — from cell surfaces, and present select few examples of intact glycopeptide mapping that provide rich information on the diversity of glycosylation of cell surface glycoproteins.

### Large scale glycoproteomic analysis

Considering a recent deluge of reviews covering all aspects of glycoproteomics 11, we instead provide a succinct overview of glycopeptide analysis evolution and point the readers toward recent focused reviews on glycopeptide enrichment 10, fragmentation 12 and data analysis 84,85.

Originally, one of the biggest challenges in intact glycopeptide analysis is the reliance on workflows originally developed for the analysis of tryptic peptides, mainly stemming from the fact that most MS instruments were built and optimized around the analysis of peptides, as opposed to glycopeptides. So, it comes as no surprise that early large-scale efforts were focused on mapping deglycosylated (PNGase F treated) *N*-glycoproteins.

In 2003 a seminal publication by Zhang et al. based on the N-glycoprotein oxidation and subsequent immobilization via hydrazide chemistry enabled large scale quantitative mapping of formerly N-linked glycopeptides providing valuable insight into glycoprotein landscape of human serum 86. Similar workflows, covered below have also become one of the key approaches for cell surface (glyco)proteome mapping. Following study by Zielinska et al. repurposed filter-aided-sample preparation (FASP) by including lectin enrichment for isolation of *N*-glycopeptides and subsequent mapping of over 6000 formerly *N*-glycosylated peptides, gaining insight into the topological constraints of N-glycosylation 87. On the other hand, the large-scale identification of intact glycopeptides was lagging, mainly because characterization of glycopeptides with collision-induced dissociation (CID) was technically challenging, and data analysis relied on a large degree of manual interpretation. Alternative fragmentation strategies such as higher energy collisional dissociation (HCD), electron-based fragmentation methods (ECD, ETD) and their combination into so called hybrid fragmentation approaches such as electron-transfer/higher-energy collision dissociation, have proven invaluable for characterization of intact glycopeptides 88-95, yielding rich fragmentation spectra that could be exploited to build better software solutions for data analysis. Indeed, over the last few years we have witnessed a small revolution in the development of numerous search engines tailored to identification and quantitation of intact glycopeptides 96-100, and we now witness studies characterizing over ten thousand on intact glycopeptides 101,102.

Finally, what is widely known, but often not stressed enough is that glycoproteomic approaches only provide a mass of a glycan attached to the peptide backbone. Actual glycan structure is often inferred based on the glycan biosynthetic pathway, and the vast body of literature knowledge stemming from the structural analysis of the released glycans serves as a reference when searching glycopeptide data 103-106. Ideally, a perfect glycoproteomic experiment would also involve parallel characterization of the glycome and *N*-glycosite containing peptides 107, a term recently popularized as glycomics-assited glycoproteomics 108. Additionally, it is worthwhile mentioning continuous advances in chemoenzymatic synthesis of glycans and glycoproteins, enabling access to libraries of structurally defined standards 109-114. Such standards have the potential to propel glycoproteomics forward through systematic investigation of fragmentation pattern of well-defined isomeric/isobaric glycan structures 115-117, akin to how libraries of synthetic peptides have advanced proteomics 118.

### Cell surface; from proteomics to glycoproteomics

Over a decade ago, oxidation and biocytin hydrazide labeling of cell surface glycans allowed the identification of formerly glycosylated peptides 119. This relatively simple but powerful method later enabled the definition of a cell surface glycoprotein atlas covering over 70 mouse and human cell lines 120. A key effort leading to the widespread use of cell surface capture (CSC) methodology is the parallel development of software tools for exploration and validation of CSC data. Notable examples here include SURFY, a machine learning-based approach for *in silico* exploration of the human surfaceome 121, and its extensive developments, providing end-to end solution from sample preparation, data analysis, validation and visualization of CSC experiments through the modular CellSurfer platform 122-124. However, the downside of the original CSC technology is the reliance on enzymatic deglycosylation of labeled glycopeptides with PNGase F, which leads to the loss of information on underlying glycans. This issue was resolved by optimizing the sodium periodate oxidation conditions to selectively modify sialic acids on the cell surface, followed by hydrazide labeling and subsequent release of the captured glycopeptides via sialic acid cleavage in mild acid conditions. Although this approach preserved most of the glycans, it resulted in limited coverage of cell surface glycoproteome 125. In addition to CSC technology, the exploitation of unique chemistries has allowed the development of a variety of glycoprotein capture techniques. Briefly, early work by Werner Reutter group reported that feeding the cells with N-acyl derivatives of N-acetylmannosamine, a metabolic precursor of sialic acid, can be used to modulate N-acyl groups of sialic acids^126^. Bertozzi group built on this research and developed a toolkit of unnaturally modified sugar analogues with azide functional groups, which get biosynthetically incorporated into glycans with the azide serving as a labelling target via copper (I)-catalyzed azide-alkyne cycloaddition, a reaction termed click chemistry for which Barry Sharpless and Morten Meldal were awarded Nobel prize in chemistry in 2022^127^,^128^. Carolyn Bertozzi then further built on this research by circumventing the toxicity of copper catalyst inventing strain-promoted azide-alkyne cycloaddition, which made these reactions compatible with living cells and animals, now known as bioorthogonal chemistry, for which she was also awarded Nobel prize in chemistry in 2022 129-132.

Alternatively, beyond metabolic labeling, researchers have exploited the promiscuity of ST6 beta-galactoside alpha-2,6-sialyltransferase 1 (ST6Gal-I) for sugar donor substrate utilizing biotinylated CMP-activated sialic acid for enzymatic biotin-labeling of cell surface glycoproteins 133. Furthermore, a number of powerful approaches building on the foundational pioneering Bertozzi/Meldal/Sharpless work have been developed and used to profile cell surface sialoglycoproteins 134, allowing analysis of changes upon drug treatment or bacterial infection 135,136. Promisingly, recent combinations of metabolic labeling and isotopic recoding have provided a blueprint for detection of glycopeptides in complex mixtures, as well as paved a way forward for large-scale quantitative exploration of the glycoproteome 137-139.

The promise of cell surface glycoprotein analysis through CSC profiling is beautifully illustrated by a large-scale comparative analysis of the regulation driven by oncogenes 140. Expression of six different oncogenes, namely EGFR, HER2, KRAS, BRAF, MEK, AKT, in isogenic epithelial cell lines demonstrated both the uniqueness in dysregulated glycoproteins and the commonalities in general biological processes such as nutrient transport, adhesion and various tumor suppressing immune modulators. Interestingly, remodeling of each oncogene could be reverted to a more common state by addition of MEK inhibitor.

Recent advances in microscale application of CSC, have reported identification of 276 141 and 800 142 glycoproteins starting from 1 million myeloma B cells, enabling the study of primary cancer cells for discovery of potential immunotherapeutic targets 142. Briefly, microscale CSC developed by Ferguson et al. enabled cell surface profiling of myeloma plasma cells leading to identification of CCR10 as a target for chimeric antigen receptor T cells and identified a panel of glycoprotein biomarkers of resistance to lenalidomide and bortezomib therapy 142. However, the current CSC technology is currently not permissive to intact glycopeptide profiling, which has the potential to uncover a whole new layer of biology as well as to identify better biomarkers and drug targets.

Beyond CSC, approaches based on differential solubility and ultracentrifugation have yielded success in characterization of intact glycopeptides from the cell surface 143-145. Park et al. study specifically evaluated the incorporation of unnatural sialic acid variants through metablolic labeling of cells with N-azidoacetyl-mannosamine (Ac4ManNAz) from plasma membrane fractions identifying over 2000 unique glycoforms in Caco-2 (Adenocarcinoma) cell line 143. Another study demonstrated impressive sensitivity, mapping thousands of glycopeptides form a limited amount of starting material, representing an opportunity for in-depth investigation of membrane glycoproteome of clinically relevant samples 145. Similarly, a sensitive stepwise glycopeptide enrichment approach based on zwitterionic hydrophilic interaction chromatography has been developed 146. The optimized protocol was used on non-small cell lung cancer identifying over 7000 intact glycopeptides from crude membrane fraction covering some of most biologically important cell surface glycoproteins such as MET, ERBB2, ERBB3, PD1L1, IGF1R, AXL, and EGFR. Notwithstanding just the receptor activation, the insights into the glycosylation of these receptors was exhaustive. For example, just in the EGFR case the authors were able to characterize 10 of 12 *N*-glycosylation sites of EGFR spanning over 150 unique intact glycopeptides paving the way for future comparative studies on the role of aberrant glycosylation at the cell surface.

Next to general exploration of cell surface glycosylation, recent recognition of glycan binding proteins, such as Siglecs and Galectins, in regulation of immune system and their potential as novel immune checkpoint inhibitors has resulted in development of approaches for targeted identification of glycan binding proteins on the cell surface^147^-^152^. Although historically considered to bind, almost indiscriminately, sialic acid on the cell surfaces, recent data point to unique ligands of siglecs such as CD43 for Siglec-7, and strikingly glycosylated RNAs expressed on the cell surfaces recognized by Siglecs 11, and 14 153,154. Along the same lines, Galectins also appear to have discriminate ligands, such as interaction of Galectin-9 with glycan at site N166 of PD-1 leading to lattice formation of Gal-9/PD-1/TIM-3 and inhibits Galectin-9 induced T-cell apoptosis 155. Recent technological developments by Lebrilla group in systematic probing of cell surface sialic-acid mediated protein by crosslinking mass spectrometry, and Huang group in proximity labeling of Galectin-3 ligands represents a promising way forward for capturing fine specificities of glycan binding proteins in living cells 150,151.

## Conclusion

The concerted community efforts on improving many aspects of the glycoproteomic workflow, from sample preparation to instrumentation sensitivity, fragmentation approaches and user-friendly data analysis software have enabled significant advancements in the field of glycoproteomics 156. As a result, we are starting to observe a rise in large scale glycoproteomics analysis of primary tissue samples. For example, glycoproteomic mapping of 119 high-grade serous ovarian carcinoma tissue samples has revealed its power in tumor molecular subtyping 156. Identification of aberrant glycosylation in cancer cells can be exploited for the development of tumor-targeting drugs 72,157 giving further support for the need to develop large scale cell surface glycoproteomics approaches. Indeed, powerful approaches have been deployed to target solid malignancies by either developing chimeric antigen receptor T (CAR T) cells that recognize aberrant cancer surface glycosylation or by removing N-glycosylation to unmask the hidden CAR T cell targets 158-160, underscoring the crucial importance of evaluating the status of glycosylation in patient derived samples 161. Additionally, glycoproteomics can be applied towards the characterization of the immune cells that combat cancer, to relate glycosylation patterns that may affect binding of ligands or signaling pathways to distinct activation or suppression states. Glycoproteomics can also assist in the design of drugs that specifically target distinct glycoforms of receptors in immune cells, potentially leading to more effective therapeutics with fewer side effects^162^,^163^.

Cell surface glycoproteomics can also find application in the discovery of new biomarkers for disease diagnosis and prediction of drug response. For example, while glycoproteomics of liquid biopsies provides a significant advantage compared to next generation sequencing approaches as it allows detection of earlier stages of disease 164, glycoproteomics of circulating cells and exosomes in blood may provide additional biomarkers 165. Similarly, while techniques like RNA sequencing have brought a wealth of knowledge about the cell types that populate the tumor microenvironment, high resolution glycoproteomics of tumor biopsies may unlock an additional layer of information that can be related to the physiological state of the cells found in the tumor microenvironment 166.

The knowledge enabled by the accurate detection of the dynamic glycan structural changes combined with peptide sequence, compared to peptide or glycan measurements alone, has enormous potential across many fields of biology and medicine. By defining the complex and diverse glycosylation patterns of surface receptors, cell surface glycoproteomics will drive significant advances in our understanding of disease pathology. We have just scratched the surface of known glycosylation and new opportunities for target identification and biomarker discovery await to be scooped from the cancer cell surface.

## Figures and Tables

**Figure 1:**
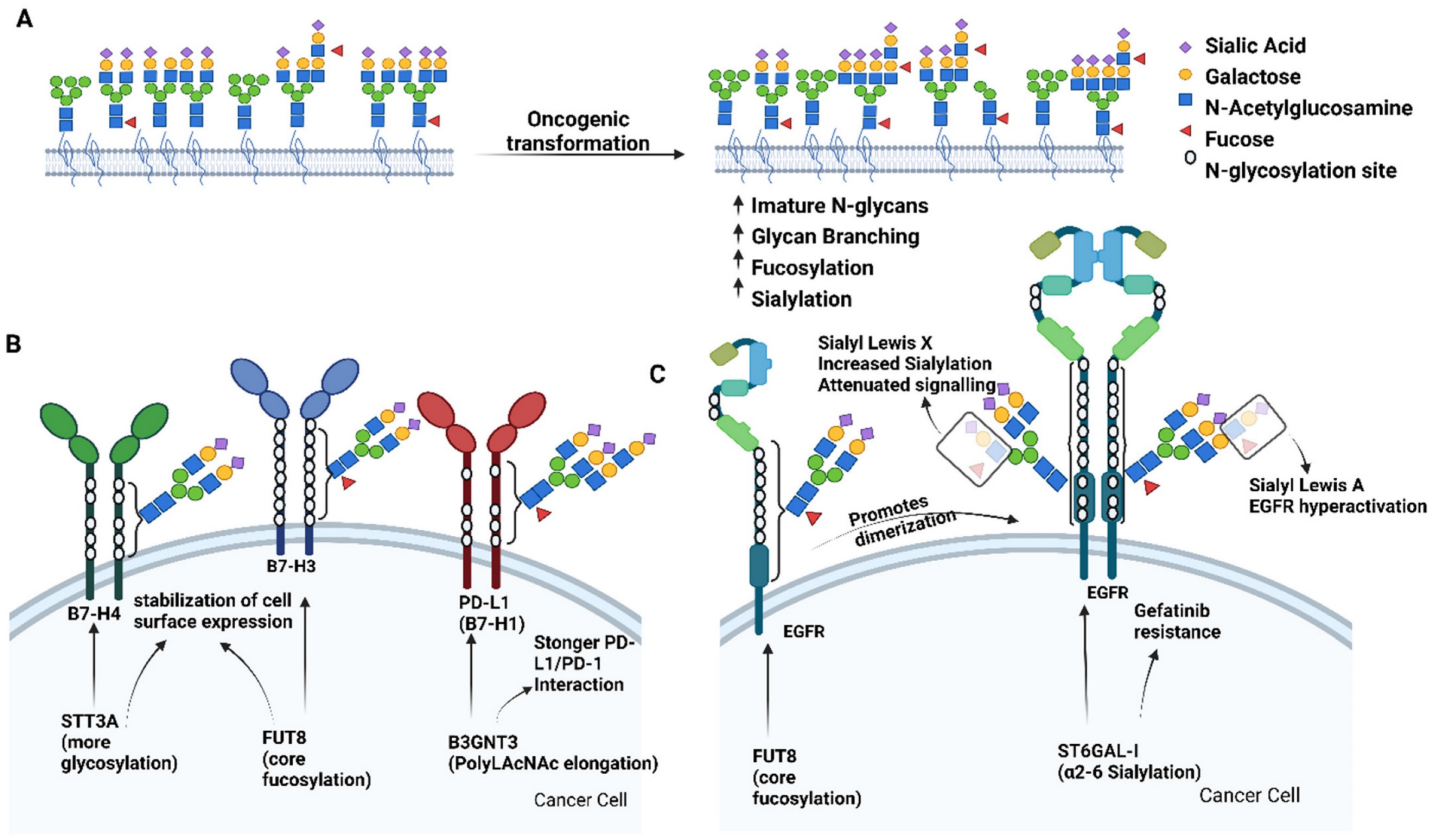
** Aberrant glycosylation in cancer**. **A**) Overview of general *N*-glycosylation changes described in cancer. Specific examples of functional consequences of aberrant *N*-glycosylation are depicted for the B7 family of immune checkpoints and Endothelial Growth Factor Receptor (EGFR) are in panels **B** and** C**, respectively. Figure created with BioRender.com.

**Figure 2:**
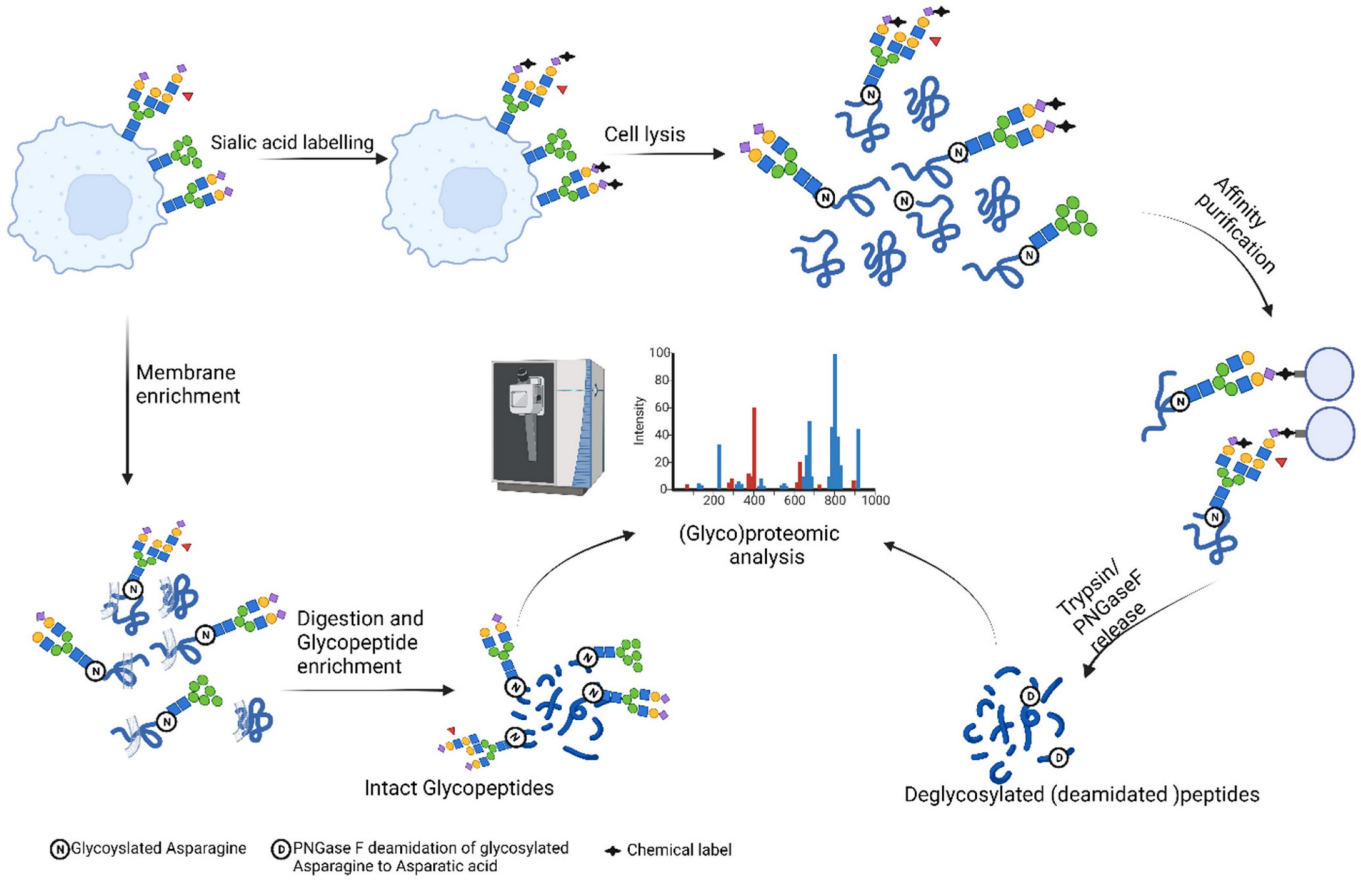
** Overview of approaches for cell surface (Glyco)proteomics.** (Created with BioRender.com).
